# The Making of “The Trafficking Problem”

**DOI:** 10.1007/s10508-018-1367-4

**Published:** 2018-12-11

**Authors:** Ine Vanwesenbeeck

**Affiliations:** 1 Department ISS: Cultural Diversity & Youth, Utrecht University, 3584 CS Utrecht, Netherlands; 2 Research Department, Rutgers, Utrecht, Netherlands

In their Target Article “The Prostitution Problem”: Claims, Evidence, and Policy Outcomes, Benoit, Smith, Jansson, Healey, and Magnuson (2018) give an excellent account of two primary positions taken toward sex work, in academia and beyond. The first position holds that prostitution is an institution grounded in gender inequality, characterized by and legitimizing the sexual exploitation of women by men. The second perspective stresses the fact that multiple forms of social inequality intersect to constitute prostitution as a form of exploited labor.

After thorough review of the available evidence, Benoit et al. (2018) conclude that the strongest empirical support is definitely for a vision of sex work as work, be it a form of notably exploited labor due to many different social inequalities. From my own experience of decades in the field (e.g., Vanwesenbeeck, 1994, 2001, 2005, 2011a, b, 2013, 2017), I fully endorse such a view. This does not, however, detract from the fact that gender inequality affects sex work practices as much as any other (sexual) practice. Sex workers are dealing with sexism as much as any other worker—woman, or man for that matter—and most probably even more, considering stigma and sex worker rightlessness. One could thus argue that there is at least some truth in both perspectives.

Benoit et al. (2018) go on to acknowledge that, while the evidence supports the “labor exploitation” over the “sexual exploitation” perspective on “the prostitution problem,” the global trend has been the opposite. This has led to “repressive policies to punish men who purchase sex, and protect women who sell sex, and to marginalize the sex sector.” Although correct to a large extent, I radically oppose an account of repressive policies as protective of women. On the contrary, much evidence now shows that repressive policies fundamentally threaten sex workers health and rights (Vanwesenbeeck, 2017). Treating sex work as exceptional, morally objectionable, and subsequently criminal is demonstrably ineffective and, as evidence shows, only makes things worse. I also have difficulty, with the one-sided causality implied by Benoit et al. (2018) as if an analysis of prostitution as a problem would lead to certain policies to solve that problem. In my view, it is repression that produces abuse and violence to a large extent and thereby causes prostitution to be “a problem” in the first place.

Although Benoit et al. (2018) do attend to these mechanisms when discussing critiques of repressive policies, they do not take a firm stand against them. Their call is foremost for more robust research that would still have to establish the validity of either of the two perspectives and the relative effectiveness of the various policy frameworks. I personally doubt the feasibility of decent comparative research as well as its usefulness. We already know that most sex work politics are morality politics and that morality politics are notably pre-scientific and evidence-resistant (Wagenaar, Amesberger, & Altink, 2017). More importantly, however, I do not think it is fruitful to go on trying to find out the “truth” about either one of two perspectives on prostitution as there is at least some truth in both of them. On the whole, I feel we should distance ourselves from this binary because it produces simplified and stereotypical images of commercial sex. Rather, we should look at the many processes behind the production of such imagery, as well of the realities of sex workers themselves. These are not stagnant phenomena. The “truth” about commercial sex is not absolute, fixed, or static. On the contrary, the sex trade is a variable, dynamic, complex world that is constantly changing and evolving. The “truth” as we know it is continually created and shaped by many different actors on many different levels, not least by the design of narratives around sex work and the implementation of certain policy regimes against the backdrop of changing global contexts, shifting economic relations, and technological developments.

Whereas Benoit et al. (2018) appear to embark on an endeavor to discover “the truth” about prostitution, I would like to shed some light on some of the subtle and not so subtle dynamics behind the construction of “the truth” and the consequences of these processes for the daily realities of sex workers. My aim is twofold: (1) to radically offset the idea of “the prostitution problem” being fixed and stable, and (2) to illustrate the determining role that repressive policies play in constituting prostitution as a problem in the first place. Specifically, I will deal with the construction of “the trafficking problem,” a notion that seems to now have become the dominant narrative about migration for sex work and even about sex work overall. I intend to illustrate that while, as Benoit et al. (2018) have shown, the overwhelming evidence is for a perspective on sex work as exploited labor, the trafficking narrative is aggressively deployed to uphold a markedly failing imagery of sex work as sexual exploitation of women and to legitimize decidedly failing repressive politics (Persak, 2014).

## Sex Workers on the Move

Many changes can be observed in the business of commercial sex of late. Due to economic, ideological, and technological developments, a diversification and professionalization of the sex industry has been noted (e.g., Cunningham et al., 2018; Ward & Aral, 2006). An increase of information and communication technologies use by sex workers and a (connected) growing worker independence are examples (Bernstein, 2007; NSWP, 2016; Sanders, Connelly, & Jarvis-King, 2016). Probably the most widely attended shift, however, is an increase in migration for sex work. Migration for work has always been commonplace, not least in the global south (e.g., van Blerk, 2008). For women, working in the caring professions in another region, country or continent has long provided a viable alternative to desperate labor situations “back home.” Sex workers have been a notably mobile crowd anyway, among others because of the stigma attached. But labor migration, not least by women, may also have increased during the last decades due to globalization and economic restructuring, the expansion of international travel, the feminization of poverty, and women increasingly seeking autonomy and independence (Agustín, 2007; Doezema, 2000; Ward & Aral, 2006). In addition, consumerism and the commodification of sex may have further enlarged the market for sex work specifically (Altman, 2001; O’Brien, 2016).

There is ample evidence that migration for sex work is, for the larger part of migrants, a deliberate, calculated choice (e.g., Agustín, 2007; Bradley, 2010; Hwang, 2017; Kempadoo, 2012; Sahni & Shankar, 2013; Weitzer, 2007, 2015). Notwithstanding the criminality and exploitation that is axiomatically part of any illegal landscape, there is, overall, little evidence of blatant force and coercion into sex work in the countries of origin (e.g., Agustín, 2006; Doezema, 2000; UN Centre for International Crime Prevention, 2003; Wagenaar et al., 2017). To the contrary, sex work migration may be a path to independence (van Blerk, 2008) and is not seldomly motivated by a level-headed flight from sex and gender-related discrimination and violence (Corrêa, Petchesky, & Parker, 2008) or by a full-fetched search for adventure and self-realization (Agustín, 2007). Hofmann (2010) coined the qualification of it being a form of bodily or corporeal entrepreneurialism, well-fitting a neoliberalist society. Evidence disclaims the stereotype that migrant sex workers are all uneducated and motivated by dire financial need (e.g., Busza, 2004; Hwang, 2017). They most certainly see themselves as agents.

However, migrant sex workers are mostly framed as devoid of agency, as vulnerable, innocent victims, ignorant, näive, and manipulable. Where sex workers in general are seen as either bad or sad, migrant sex workers are seen as mainly sad. There seems to be a widespread, fundamental inability to combine considerations of push and pull factors for migration with an acknowledgement of migrants’ agency (Agustín, 2006). Ethnocentrism and a Western or even colonial gaze may be guilty here (cf. Doezema, 2000; Kempadoo, 2012), but a good dose of sexism may be at work as well: men migrate, women are trafficked. And not to be understated, a programmatic, strategic maneuvering by a huge anti-trafficking lobby systematically promotes these negative qualifications as part of their moral crusade against prostitution.

## Anti-Traffickers on the Move

It has been broadly documented that attention for sex trafficking has skyrocketed during the last couple of decades (Agustín, 2006; Bernstein, 2014; Bradley, 2010; Kempadoo, 2012; Weitzer, 2007; Wijers, 2015). Magnanti (2016) gave a telling illustration by calculating from news database searches that there were only three references to “human trafficking” before 2000, more than 500 in 2010, and tens of thousands since then. The United Nations *Trafficking Protocol to Prevent, Suppress and Punish Trafficking in Persons* launched in 2000, and the ensuing War on Trafficking by the Bush administration have been crucial in this respect. At present, the anti-trafficking movement consists of a diverse coalition of conservative (evangelical) Christians, fundamentalist Islamists, abolitionist feminists, social activists of diverse stripes, a cadre of Hollywood celebrities and corporate officials (cf. Bernstein, 2014; O’Brien, 2016). In the U.S. alone, possibly hundreds of ideology-based organizations, often privately funded with huge sums of money by right-wing, conservative evangelicals, now exclusively devote themselves to the war against trafficking, which many admit is really a war against prostitution (Magnanti, 2016).

The global “anti-trafficking juggernaut” (Kempadoo, 2012) has been analyzed against the backdrop of “the return of the religious,” notably the reactive politicization of conservative Christianity (Bernstein, 2010; Corrêa et al., 2008, Corrêa, De la Dehesa, & Parker, 2014), as well as of the countermovements of globalization as expressed in strong nationalism, populism, and anti-migration sentiments (Corrêa et al., 2008; Kempadoo, 2012). Commentators (e.g., Doezema, 2000) observe parallels with the moral panics and white slavery debates in the beginning of the late nineteenth and early twentieth centuries, where deeper fears and uncertainties relating to women’s growing mobility and power, a downfall of the family, a loss of national identity, increasing migration, and perceived threats to social stability also played a major role. The geographical direction was different but the same rhetoric is employed, and both must be unmasked as cultural myths, Doezema (2000) asserts. As sexual rights gain visibility, homophobia escalates (Corrêa et al., 2008) and whorephobia probably no less. The “rise of the social” in which a newly empowered bourgeoisie felt qualified to rehabilitate inferiors (Agustin, 2007) and an overall NGO-ization in international cooperation (Bernstein, 2014; Corrêa et al., 2008) have also been added to the equation that has resulted in a renewed, powerful, worldwide anti-prostitution movement.

## The Making of “The Trafficking Problem”

The anti-prostitution movement makes a good many claims, such as violence being omnipresent in prostitution, customers and traffickers being the personification of evil, and sex workers lacking agency, which may all be exposed as problematic, unsubstantiated, and/or false (cf. Weitzer, 2007, 2015). Crucially, they principally reject the very concept of benign migration for sex work. One of the strategies is to present only the horror stories and atrocity tales. Terms such as “prostituted women” and “sexual slavery” are employed as “staples in their discourse” (Weitzer, 2007, p. 451) and make any agency in migrant sex workers invisible in the first place. One central, evidently unsubstantiated and seemingly false claim is about the magnitude of violent trafficking as “greatly increased” and “very high” with “thousands of trafficked women” existing at “an epidemic level.” Figures used are incredibly elastic, ranging from some hundreds thousands of trafficked victims to three or even four million annually. And many media outlets report such figures matter-of-factly.

However, the production of these high numbers has been critiqued for a lack of methodological transparency and source documentation, for incorrect extrapolations, and for unacceptable broadening of the definition of what constitutes a victim (e.g., Gozdziak & Collett, 2005, for the U.S.; Kelly, 2005, for Europe). Inflation of numbers takes place by, for instance, defining all underage persons as trafficked. Also, the term sex trafficking is often used for all migrants or “every instance of relocation to a destination where an individual sells sex” (Weitzer, 2007, p. 453). The definition has also been expanded in such a way that sex workers’ regular practices of sharing space and sharing information, either online or offline, are considered trafficking (e.g., Burns, 2015). Other strategies include the expansion of the number of victims with all “youth at risk of violence” or with all “possible victims,” qualifications strongly subject to subjective opinion and profiling. Yet another strategy is to disguise all trafficking as sex trafficking. As Magnanti (2016) notes, the International Labor Organization estimates that no more than 21% of trafficked people are in forced sexual labor, but “when you listen to the media you wouldn’t think trafficking comes in flavors other than sex” (p. 51). As a consequence of such clear distortions, an embarrassing discrepancy has arisen between extremely high numbers claimed for victims of trafficking (up to 50,000 every year) in the U.S. and the really low number (less than 2000 *in 10* *years*) of T-visas (visas specifically for victims of trafficking) granted (Bernstein, 2010). Magnanti (2016) sniggers that, on Twitter, there are more accounts for groups supposedly raising awareness of trafficking than there are *actual documented victims* (italics in original) (p. 71).

Enormous amounts of money are spent on efforts to prove sex trafficking and find victims. Magnanti (2016) provides a series of accounts of such unfruitful endeavors. For instance, the FBI’s “Operation Cross Country” contributed something in the order of $40 million in 2015 and 2016 to fight trafficking of children, resulting in the grand total of two arrests of suspected traffickers, each with one victim, a 16-year-old and a 17-year-old (p. 60). In the UK in 2007, a wide-scale investigation where 55 police forces used every method at police disposal (1300 locations were raided, 255 women—who later turn out to be voluntary migrants—were “rescued”) resulted in five convictions of men across the country (Magnanti, 2016, p. 55). Another example is the Polaris Project in the U.S., which oversees the National Human Trafficking Resource Centre’s telephone hotline. It obtained $3.2 million in funding in 2010, during which time they received 471 calls, which boils down to about $7000 per call. Magnanti (2016) cites fundingtrends.org, who report that funding for studying trafficking rose from almost nothing in 1991 to the tune of 600 million US dollars by 2010. By the end of 2016, the value of grants awarded for trafficking amounted to double the money offered for lung cancer and 20 times greater than the funding for malnutrition, malaria, and tuberculosis combined. It has been calculated that the 50 largest anti-trafficking organizations in the U.S. have an estimated income of about $700 million per year, as of 2015. Combined with another 40 anti-trafficking groups funded by the U.S. State Department, budgets top $1.2 billion. “138 traffickers were convicted in 2012: that’s $8.6 million, minimum, per conviction,” Magnanti (2016) again poignantly observes (p. 73).

The lack of evidence does not seem to bother the anti-trafficking lobby, nor its financers, or authorities. To the contrary, in many places, the anti-prostitution ideology has become state policy, in the U.S. for one. Weitzer (2007) illustrated how the anti-prostitution crusade has permeated every level of policy processes. Crusade ideology is officially recognized and endorsed; many programmatic and legal changes are in accordance with the ideology. Sex worker rights activists on the other hand (derogatively called the “pro-prostitution-lobby” by the anti-trafficking movement), totally lack such access to U.S. state elites. The popularity of the anti-trafficking rhetoric among state officials has also been linked to recent neoliberal social transformations of “the state” itself: a solid strengthening of carceral politics, and new techniques of surveillance and social control (Bernstein, 2014, p. 348). Radical feminism has also become increasingly “carceral,” not least in their anti-prostitution lobby.

## “Americanization” of Sexual Politics

Clearly, the influence of the U.S. reaches far beyond its own borders. The U.S. footprint on global developments in matters of sex work, and sexual and reproductive health and rights more generally, is huge (cf. Corrêa et al., 2008; Kempadoo, 2012). Reviewing the evidence, Altman (2001) already pondered whether globalization should not rather be called “Americanization.” At that time, the Global Gag Rule (GGR: a U.S. foreign policy that prohibits foreign NGOs that receive U.S. family planning funds from providing or advocating for abortion) had just been reinstated by the Bush administration. The GGR has ostensibly served as a barrier to a wide range of health services for women and girls globally (e.g., Center for Health and Gender Equity, 2017). A few years later, Bush launched the President’s Emergency Plan for AIDS Relief (PEPFAR), one of the largest and most influential donor programs in this area that strongly promoted abstinence and was notably ineffective in serving sex workers (Pisani, 2008). The Bush administration also cut funding for The United Nations Family Planning Association, a key source of financial assistance for reproductive health programs. These measures had disastrous consequences for the altogether vulnerable sexual and reproductive health infrastructure in many developing countries (Vanwesenbeeck, 2011b). The global impact of Bush’s prescriptions on sex have been widespread and deep. They inspired conservative states and social forces to push their political and religious agendas and, in a large number of cases, Bush administration guidelines and prescriptions have been incorporated into national policy frames (Corrêa et al., 2008, p. 39). And then there is also the invasion in Iraq, which fueled anti-Western sentiments in the Middle East and the Arabic world, promoting the rise of a fundamentalist version of Islam that holds notably negative views on prostitution (Bradley, 2010).

Two U.S. initiatives directly targeted sex work. In 2003, the Anti-Prostitution Loyalty Oath, also known as the anti-prostitution pledge, was put in place as part of PEPFAR and requires NGOs that receive federal anti-HIV/AIDS or anti-trafficking funds to adopt an organization-wide policy opposing prostitution and sex trafficking. This provision has had many negative ramifications in the fight against AIDS and, notably, for sex workers’ health and rights worldwide (e.g., Center for Health and Gender Equity, 2015). In addition, after the Trafficking Victims Protection Act was put in place in 2000, national interventions to combat trafficking (except their own) are now evaluated by the US State Department annually. Countries are ranked and those that do not comply with U.S. standards are placed in the lowest tier and subject to sanctions. The lowest tier mainly consists of countries that the U.S. considers to be unruly anyway, illustrating that the trafficking discourse is used toward political ends rather than to protect victims (e.g., Kempadoo, 2012). Nevertheless, many countries try to comply, in an effort to compete for international recognition (Bernstein, 2014). Ironically, national anti-trafficking measures have not only increased abuse of sex workers and human rights violations (e.g., Corrêa et al., 2014; GAATW, 2007; Vanwesenbeeck, 2017), but also stimulated rather than reduced migration among sex workers (Ditmore, 2008; Hwang, 2017).

U.S. abolitionists have demonstrably put in a lot of effort to insert their anti-prostitution framework into the international arena. Europe has certainly been targeted fiercely and with huge sums of money (Hoff, 2014). A leading role has been taken on by Sweden that has (as part of their ongoing “nation branding”) aggressively marketed its “Swedish model” of client criminalization as a feminist policy par excellence (Florin, 2012; Outshoorn, 2015). A notable low among the consequences was the European Parliament endorsing an advisory motion promoting “demand reduction” among its member states in 2014, while in 1986 the Parliament recommended decriminalization of the “exercise of the profession” (Euchner & Knill, 2015).

## The Workings of Anti-Trafficking Politics

Although the anti-trafficking lobby’s main tenet is protection of victims, the evidence that anti-trafficking policies and criminalization of sex work achieve just the opposite is overwhelming. Repressive sex work regimes are at serious odds with human rights and public health principles. Repression fuels stigma and all its negative consequences. It escalates sex workers’ risks and vulnerabilities and negatively impacts working routines and relations. It reduces access to health care and the legal system, denies sex workers’ authority and self-determination, and blocks ways out of the industry (Vanwesenbeeck, 2017). Underage sex workers in the U.S. “rescued” in “the fight against trafficking” do not get salvation but get detention, court fees, and criminal records that only make their lives more difficult (Moore, 2015). Arbitrary arrest and detention, displacement and forced rehabilitation, deportation, and denial of access to justice are among the many human rights violations documented as a consequence of “the war on trafficking” (e.g., Agustín, 2007; Amnesty International, 2016; GAATW, 2007; ICRSE, 2016). Empower Foundation (2012) in Thailand suggested that rescuers actually posed a greater threat to the safety of sex workers than traffickers. I stand with Magnanti (2016) who concludes: “The closer we look at the truth about trafficking, the more we find not women and children being saved from terrible fates, but powerful agencies claiming money and attention for themselves while the people they supposedly rescue are arrested, deported, and fall through society’s cracks” (p. 49).

As a rule, migrant sex workers are hit the hardest. Everywhere, their social and working positions are often weak and vulnerable. When ending up in anti-trafficking raids, migrant women are the first to be violated. They are routinely “out-migrated” without any protection being offered. Migrant sex workers are seen as victims but treated as criminals, Kempadoo (2012) observes. However, actual victims of trafficking are notably ill-supported. In contrast to the huge sums of money being spend in “the war on trafficking,” progress in the area of victim assistance and conviction of traffickers is notably slow (Dottridge, 2014). Restrictive anti-trafficking measures fail to support victims, violate human rights, threaten health and safety, and actually drive migrants into the arms of exploitative brokers (Hwang, 2017). Anti-trafficking awareness campaigns construct a notably narrow understanding of the problem by depicting “ideal criminal offenders” and they obscure the role restrictive migration regimes in the destination countries play (O’Brien, 2016). As a result, migrants are rendered vulnerable to traffickers instead of being “saved” from them.

## Conclusion

The radical feminist position that sex work is, by definition, a form of violence against women has grown into the proposition that all sex work is, by definition, a form of trafficking. Sex work policies have been reduced to morality-based policies against trafficking, with ample attention to restrictions on migration. Anti-trafficking politics target prostitution as the problem, not the concrete problems and inequalities in and behind prostitution that lead to sexual violence and (labor) exploitation in their many forms. Maybe Benoit et al. (2018) started on the wrong foot by looking at perspectives on “the prostitution problem” in the first place. As much as “the trafficking problem,” use of the phrase “the prostitution problem” contributes, in its negativity, one-sidedness, and lack of nuance, to unrealistic cultural myths about sex work. There is, however, nothing mythical about the actual exploitation and abuse of women (and men) in sex work. But framing the whole industry as “the problem” is fueling stigma and its consequences. It forecloses effective strategies to fight exploitation and assist its victims. Dottridge (2017), an ex-director of Anti-Slavery International, makes this point on the framing of sex work as Modern Slavery: “The types of exploitation implied by Modern Slavery encourage many government officials to stop paying attention to conventional techniques for protecting workers such as regulation, workplace inspections, and trade unions. By creating the impression that they are helpless slaves who need rescuing from the hands of criminals, they propagate a myth that all informal work that helps migrants to survive is illicit and should be prohibited, thereby denying migrants the lifeline on which they often depend.” Demonstrably effective strategies to fight and prevent labor exploitation in sex work, encompass full decriminalization, community building integration, and collective governance (cf. Amnesty International, 2016; Östergren, 2017; Vanwesenbeeck, 2017; Wagenaar et al., 2017). And any effective fight against exploitation should involve the workers themselves, not dismiss them. Fortunately and increasingly, sex workers do claim voice and will not let themselves be silenced any longer (e.g., GAATW, 2018; Mgbako, 2016; Stevenson & Dziuban, 2018). Hundreds of organizations worldwide are now members of the umbrella Global Network of Prostitution Projects and a couple dozen more are estimated to operate more or less independently. Sex workers’ migration networks have also been formed (Hwang, 2017). The representation of the sex worker movement in policy making is hampered by a notion of prostitution as “the” problem. Sex workers should not be seen as part of the problem but be acknowledged as essential and valuable partners in the solution.
